# Anaphylaxis to gadobutrol and gadoterate meglumine in a patient with low grade astrocytoma

**DOI:** 10.1186/2045-7022-4-S3-P6

**Published:** 2014-07-18

**Authors:** Juan Manuel Escobar Montalvo, Ruth Mielgo Ballesteros, Ruth Barranco Jimenez

**Affiliations:** 1 Hospital Universitario 12 De Octubre, Allergy Service, Madrid, Spain

## Background

Gadolinium is a contrast agent considered as safe for magnetic resonance imaging (MRI). We report a patient with a low grade astrocytoma who had two anaphylactic reactions after administration of Gadobutrol and Gadoterate Meglumine respectively in a routine brain MRIs.

## Method

A 58-year old woman with low grade astrocytoma, who had previously tolerated Gadolinium-based contrasts, was diagnosed with anaphylaxis to Gadobutrol by history of anaphylaxis after intravenous administration and positive skin testing. For these reasons Gadobutrol administration was banned and was given a premedication protocol (steroids and antihistamines) if the patient will require future administrations of other Gadolinium-based agents. However, one year later, despite premedication the patient presented with pruritus and hives disseminated, breathlessness, dizziness and hypotension two minutes after administration of Gadoterate Meglumine in a control brain MRI. Skin testing with Gadoterate meglumine, and Gadopentetic acid was carried out. Histamine and isotonic saline were used as positive and negative controls respectively.

## Results

The results of skin testing (prick and intradermal) with Gadoterate Meglumine and Gadopentetic acid were negative. Histamine and isotonic saline were used as positive and negative controls respectively. Intravenous challenge with Gadoterate meglumine was not performed by the suggestive clinical diagnosis of anaphylaxis and history of Gadobutrol anaphylaxis (IgE-mediated). For the severity of reactions, future administrations of Gadolinium-based contrasts were banned in the patient.

## Conclusions

We present a case of IgE-mediated anaphylaxis to Gadobutrol and anaphylactic reaction with Gadoterate Meglumine in a patient with low grade astrocytoma. The anaphylactic reactions to referred contrasts could be explained by cross-reactivity.

